# Effect of Stress on Irradiation Responses of Highly Oriented Pyrolytic Graphite

**DOI:** 10.3390/ma15103415

**Published:** 2022-05-10

**Authors:** Zhihan Hu, Di Chen, SeungSu Kim, Rijul Chauhan, Yongchang Li, Lin Shao

**Affiliations:** 1 Department of Nuclear Engineering, Texas A&M University, College Station, TX 77843, USA; cirtus@tamu.edu (Z.H.); gogottm@tamu.edu (S.K.); chauriju@tamu.edu (R.C.); ycli@tamu.edu (Y.L.); 2 Department of Physics & Texas Center for Superconductivity, University of Houston, Houston, TX 77204, USA; dchen33@central.uh.edu

**Keywords:** radiation damage, graphite, molecular dynamics simulation, Raman

## Abstract

The effect of stress on irradiation responses of highly oriented pyrolytic graphite (HOPG) was studied by combing molecular dynamics (MD) simulation, proton irradiation, and Raman characterization. MD simulations of carbon knock-on at energies < 60 eV were used to obtain average threshold displacement energies (${\bar{E}}_{d}$) as a function of strain ranging from 0 to 10%. Simulations at a higher irradiation energy of 2–5 keV were used to study the effect of strain on damage cascade evolution. With increasing tensile strain, ${\bar{E}}_{d}$ was reduced from 35 eV at 0% strain to 31 eV at 10% strain. The strain-reduced ${\bar{E}}_{d}$ led to a higher damage peak and more surviving defects (up to 1 ps). Furthermore, high strains induced local cleavage around the cavities, as one additional mechanism of damage enhancement. Experimentally, HOPG film was folded, and the folded region with the maximum tensile stress was irradiated by a 2 MeV proton beam. Raman characterization showed significantly enhanced D to G modes in comparison to the stress-free irradiation. Based on the strain dependence of ${\bar{E}}_{d}$ and the Kinchin–Pease model, a formula for displacement estimation under different tensile strains is proposed. The stress effects need to be considered in graphite applications in a reactor’s harsh environment where both neutron damage and stress are present.

## 1. Introduction

Irradiation effects in graphite are important for nuclear reactor applications. Graphite has been used as a neutron moderator and reflector in various reactor designs, from its first usage in Chicago Pile 1 back in 1942 to its proposed usage in Generation IV concepts [1]. Graphite exhibits unique irradiation responses that cause unusual thermal, mechanical, and creep property changes [2]. Neutron-induced interstitials can significantly increase the energy of graphite. If the irradiation temperature is above a critical value, the defects can rearrange themselves. Otherwise, the stored energy due to interstitial accumulation, called Wigner energy, can be suddenly released and causes a local temperature spike [3]. Reactor grade graphite exhibits a complicated radiation-induced dimensional change, including shrinkage at the beginning, then turnaround, swelling, and cross-over at higher damage levels [2,4]. The critical dose at the turnaround shifts as a function of temperature. It is believed that the initial shrinkage is due to the defect interactions with pre-existing Mrozowski cracks and porosity, which absorb carbon displacements [5].

As all property changes evolve as a function of damage level, it is important to accurately predict displacement creation under various extreme conditions. One such extreme condition is stress. Residual stress exists in as-manufactured components of complicated geometry, and the stress is expected to change during applications considering temperature difference, neutron flux difference, and irradiation-induced dimensional changes. The local shrinkage at a low damage level is expected to introduce local tensile stress and shear stress. Understanding the stress effect in damage creation is the first step to develop a predictive capability in describing graphite evolution.

The quantitative characterization of point defects in carbon materials has been challenging. Scanning tunneling microscopy is limited to small area and surface characterization [6]. For bulk materials, the traditional methods, such as channeling Rutherford backscattering, is difficult due to large minimum yields caused by polycrystalline structure, even in high-quality HOPG [7]. Raman, on the other hand, has been widely used to indirectly characterize defects in various carbon materials, including graphene, carbon nanotubes, and graphite [8,9,10,11]. The intensity ratio change of the D to G Raman modes is linked to a defect population through a modified Tuinstra–Koenig (TK) model [8]. The D enhancement effect at low damage levels is caused by reduced crystal sizes due to defect introduction. At high damage levels, if the neighboring defect distance is comparable to the six-fold ring size, the number of D active centers is reduced, and the D mode intensity decreases. Such a transition occurs when graphite/CNT/graphene becomes amorphized. In CNT, this threshold damage is about 0.17 dpa (displacements per atom) [8]. Using electron diffraction analysis, the threshold damage for amorphization is about 0.2 dpa in graphite under carbon self-ion irradiation, and the value increases to 0.5 dpa for electron irradiation at room temperature [12].

Molecular dynamics (MD) simulations have been used to understand atomic-scale details of radiation damage creation in carbon systems [13,14,15,16,17,18], but studies on graphite are relatively limited. One issue in MD simulations of carbon systems is how to correctly include long-range inter-plane interactions. Smith used the Tersoff potential to simulate the sputtering of graphite and diamond under ion bombardment [19,20]. Later, the study was extended to various carbon interatomic potentials for comparison [21]. The threshold displacement energy, averaging over all possible directions, was reported to be 34 eV for AA stacking and 34.5 eV for AB stacking in graphite [21], using the second parameterization of Brenner’s hydrocarbon potential [22]. The Brenner potential was used since it gives a better description of the C_60_ structure. Both the Tersoff potential and Brenner potential use a short-range cutoff, which may cause a problem if inter-planar interactions need to be considered. Nordlund et al. later modified the Tersoff potential by including a long-range extension [23]. The modified potential was able to show hillock formation on graphite surfaces [23]. A density function theory-based first-principles molecular dynamics study was used to study early-stage defect evolution under irradiation [24]. Although first-principles calculations lack a correct description of weak Van der Waals interactions, the problem can be mitigated. Various point defects and small defect configurations were identified in irradiated graphite [24]. The adaptive intermolecular reactive empirical bond order (AIREBO) potential was developed to include inter-layer covalent bonding and Van der Waals forces [25]. The AIREBO potential has been used in various ion irradiations and molecular irradiations of carbon materials [14,16].

Although numerous studies have been performed to obtain threshold displacement energy, the value ranges from 10 eV to 70 eV, from various experimental and modeling studies, as reviewed by Banhart and Zinkle [26,27]. The effect of stress on threshold displacement energy, however, has not yet been reported. In the present study, we used MD simulation to study the displacement creation under different levels of tensile strain. As a way to indirectly validate the findings, we combined proton irradiation, graphite folding, and Raman characterization to compare irradiation responses of graphite with and without tensile stress.

## 2. Modeling Procedure

**Figure 1:**
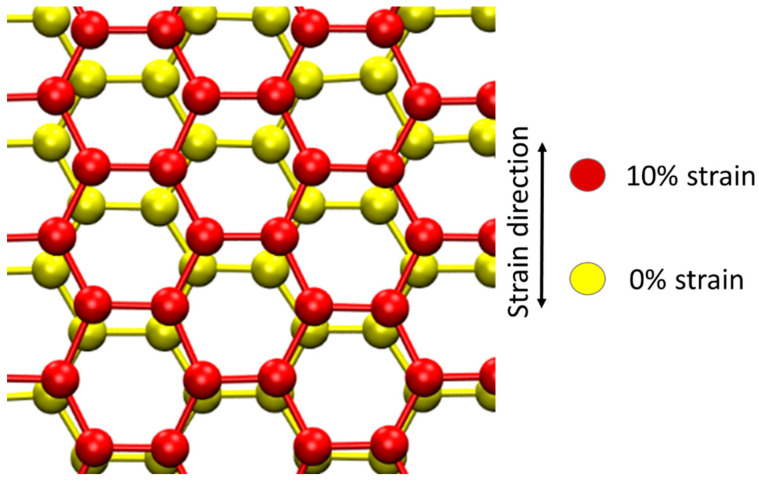
Schematics of graphite cells with and without 10% strain. The arrow points to the stretching direction.

MD simulations were performed using the Large-scale Atomic/Molecular Massively Parallel Simulator (LAMMPS) [28]. The carbon interatomic potential was described by the AIREBO potential [25]. For the determination of the average threshold displacement energy, a hybrid potential (AIREBO potential + Tersoff/ZBL potential) was included for comparison [29]. The cell dimensions were 136 Å × 246 Å *×* 213 Å and contained 800,000 atoms. A timestep of 0.2 femtoseconds (fs) was used for collision. A timestep of 2 fs was used for relaxation. The cell had a periodic boundary condition in all directions. The structural relaxation step lasted 20,000 fs at 300 K. For the determination of the average threshold displacement energies, 1000 atoms in the cell were randomly selected as projectiles of initial kinetic energy ranging from 10 eV to 60 eV. The displacing direction was random. For studying the damage cascade evolution, only four atoms in the cell were displaced, with the initial kinetic energy ranging from 2 keV to 5 keV. No damage cascade overlapping was allowed. The cell was stretched at a strain of 0, 2%, 5%, 8%, and 10% to study the stress effects. For each strain and energy condition, at least 20 ion bombardments were simulated. Figure 1 shows the graphite cells with or without 10% strain. The arrow points to the stretching direction to introduce the strain.

## 3. Experimental Procedure

HOPG samples were irradiated at room temperature by 2 MeV protons using a 3 MV NEC tandem accelerator. The beam spot size was 5 mm × 5 mm and it was rastered over an area of 11 mm × 11 mm. The beam current was 260 nA, which was intentionally controlled to be low to minimize beam heating. The proton fluence was 1.3 × 10^17^ H^+^/cm^2^. The vacuum during the irradiation was 6 × 10^−8^ torr or better.

Raman characterization was performed on four different samples: pristine HOPG, proton-irradiated HOPG, folded → proton-irradiated → unfolded HOPG, and folded → unfolded HOPG without irradiation. The folding was used to introduce tensile stress during the irradiation. The comparison between the folded → irradiated → unfolded sample and the folded → unfolded sample without irradiation was used to evaluate the irradiation effect only. Raman spectroscopy was performed using a Horiba Jobin-Yvon LabRam microscope, with a He-Ne laser at a wavelength of 633 nm, as well as a stigmatic 800 nm spectrograph with two confocal spectrometer entrances, one connected to the microscope, the other via a fiber optics coupler. The excitation light was focused with an objective 10×. The scattered light was recollected and focused onto a 200-μm-diameter pinhole. The beam cross-sectional diameter was 75 μm. The laser power was 95 μW. For each sample, three spectra at slightly different locations were collected. These spectra were found to be very similar to each other. Hence, one typical spectrum of each sample was reported.

HOPG was purchased from SPI Supplies Inc. (West Chester, PA, USA). Each HOPG was 3 mm in diameter and 50–75 µm in thickness. The mosaic angle was 3.5° ± 1.5°. One HOPG was pasted on a flat surface for irradiation. The other HOPG was folded in half, and the folded/curved region was exposed to the beam. After the irradiation, the sample was unfolded and characterized by Raman spectroscopy.

## 4. Molecular Dynamics Simulations

**Figure 2:**
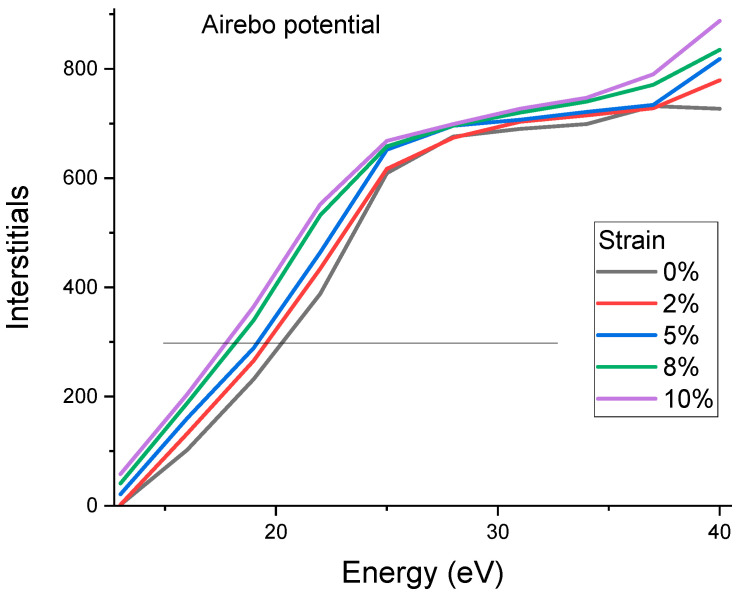
Numbers of interstitials created by carbon knock-on as a function of initial kinetic energies. Simulations were obtained using AIREBO potential.

Figure 2 plots the interstitial numbers as a function of carbon knock-on energies. The energies were internationally low to create Frenkel pairs instead of damage cascades. The energy was incrementally changed from 10 eV to 60 eV, in steps of 3 eV. The defect numbers first increase rapidly with increasing energies and then approach a plateau region, followed by a quick rise at higher energies. The curve follows the expectation from the Kinchin–Pease Model [30]: the defect creation efficiency was one at energies of $E_{d}<E<2E_{d}$ and was 0 for $E<E_{d}$. However, the curve deviates from a rigid step-like profile because (1) the threshold displacement energies are directionally dependent and the varying *E_d_* values shift the profile, leading to gradually rising fronts; (2) thermal vibration also contributes to a similar shifting effect. The total carbon knock-on numberis 1000, but the height of the plateau region is about 700, suggesting that about 30% of the defects are recombined. The defect creation is reduced to zero at the minimum $E_{d}$. However, the average ${\bar{E}}_{d}$ is more meaningful in predicting defect creation. Figure 2 shows a clear stress effect in which the defect creation curves appear to shift to lower knock-on energies due to the reduction in the threshold displacement energies under stress. The line in Figure 2 refers to the half-height of the plateau region and is used to measure the ${\bar{E}}_{d}$. For 0%, the ${\bar{E}}_{d}$ is measured to be 20.3 eV. The values decrease with increasing stress consistently. For 10% strain, the ${\bar{E}}_{d}$ decreases to 17.8 eV.

**Figure 3:**
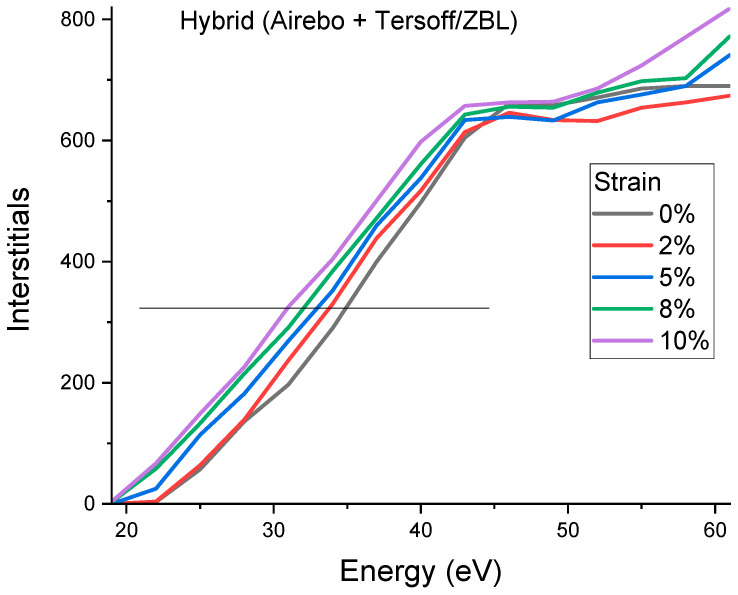
Numbers of interstitials created by carbon knock-on as a function of initial kinetic energies. Simulations were obtained using the hybrid potential (AIREBO + Tersoff/ZBL). The horizontal line is used to measure the average $E_{d}$.

The AIREBO potential was introduced to increase the accuracy in describing long-range interactions [25]. For short-range interactions that are important to collisions, the Tersoff potential linked with the Ziegler–Biersack–Littmark (ZBL) potential at a short distance may be more appropriate [29]. For comparison, the modeling was repeated using a hybrid AIREBO + Tersoff/ZBL potential. Figure 3 plots the modeled defect creations as a function of knock-on energies using the hybrid potential. The overall curve shifting under different stress is similar to that obtained using the AIREBO potential, except the starting ${\bar{E}}_{d}$ at 0% strain is much higher. The hybrid potential obtains 35 eV, which is in good agreement with previous studies (34 to 35 eV was reported in references [22]).

**Figure 4:**
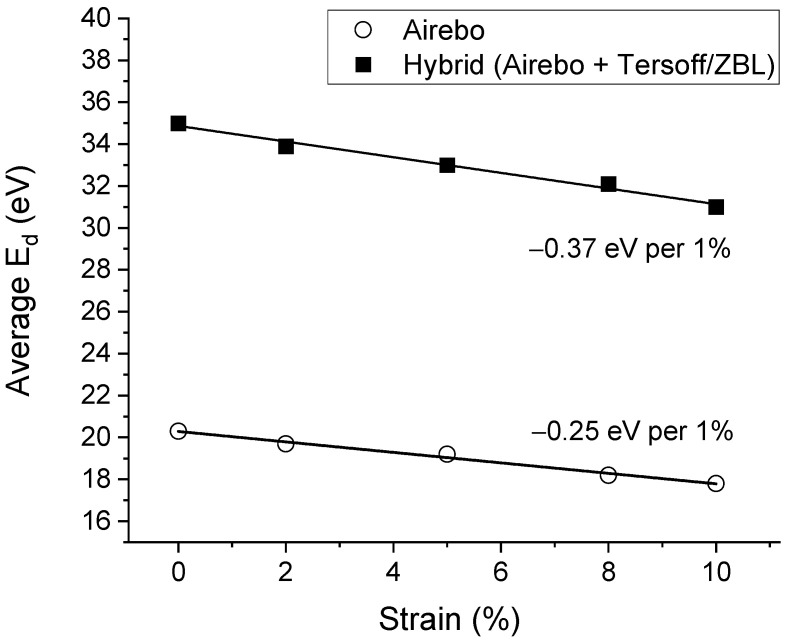
Simulation-obtained ${\bar{E}}_{d}$ values as a function of strain, calculated using the AIREBO potential and the hybrid potential (AIREBO + Tersoff/ZBL).

In summary, Figure 4 plots the ${\bar{E}}_{d}$ changes as a function of stress obtained from the AIREBO potential and from the hybrid potential. The two potentials create almost two parallel lines, with the largest difference from the ${\bar{E}}_{d}$ at 0% strain. The effects of strain on the ${\bar{E}}_{d}$ changes, characterized by the curve slope, are comparable. The change rate was −0.37 eV per 1% strain from the hybrid potential and −0.25 eV per 1% strain from the AIREBO potential.

**Figure 5:**
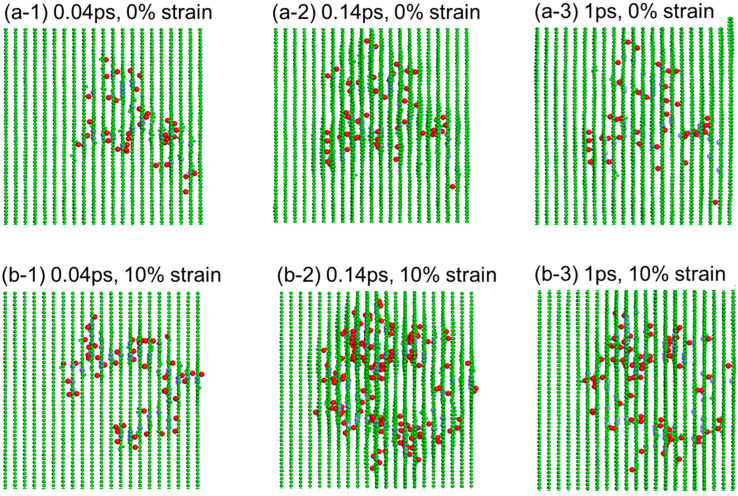
MD simulations of graphite bombarded internally by one 3.7 keV C atom at different times for (**a**) 0% strain and (**b**) 10% strain. The red refers to the C interstitials and the purple refers to the C vacancies. The green refers to the C lattice atoms.

The strain impacted both defect creation and damage cascade evolution. The latter was simulated using higher knock-on energies. Figure 5a,b shows the damage cascade evolution at the times of 0.04 ps, 0.14 ps, and 1 ps, for 0% strain, and 10% strain, respectively. The red color refers to the carbon interstitials. The green color refers to the lattice atoms. The cell is viewed along the basal planes to highlight the interstitials. The basal planes hide the appearance of C vacancies (purple color). The time of 0.14 ps corresponds to the time creating the maximum displacements for 10% strain. At a longer time of 1 ps, the defect numbers decrease due to defect recombination. The number of surviving defects, although reduce s at a longer time, is higher in the stressed cell.

**Figure 6:**
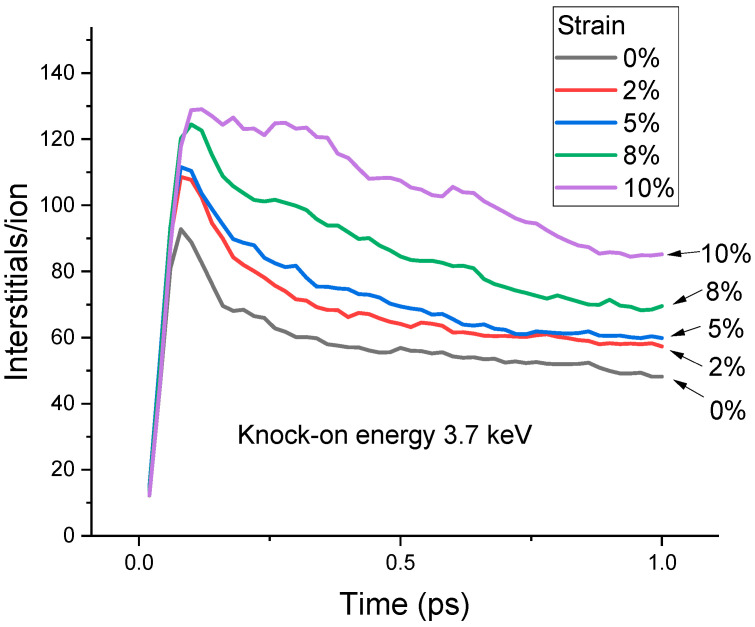
Interstitial numbers as a function of time for different strains. The knock-on energy is 3.7 keV.

Figure 6 plots the displacement number changes as a function of time under different strains. The curves represent the statistical average from >20 individual bombardments under each strain. The displacement peak heights increase with increasing strains. The maximum interstitial numbers are 92, 108, 112, 124, and 130 for 0%, 2%, 5%, 8%, and 10% strain, respectively. The surviving defects at the end of 1 ps also increase with increasing strain. The interstitial numbers are 48, 56, 59, 69, and 85 for 0%, 2%, 5%, 8%, and 10% strains, respectively.

**Figure 7:**
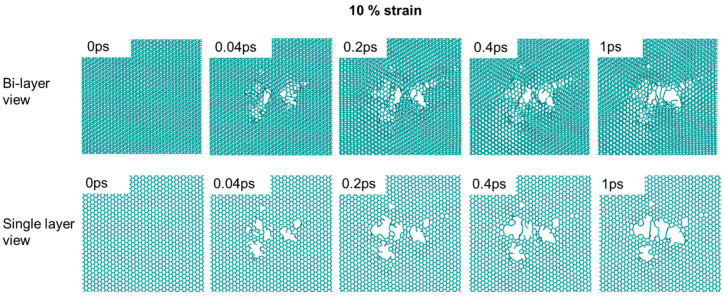
Damage cascade evolution in 10% strained graphite, viewed as a bi-layer and as a single layer. The carbon knock-on energy is 3.7 keV.

**Figure 8:**
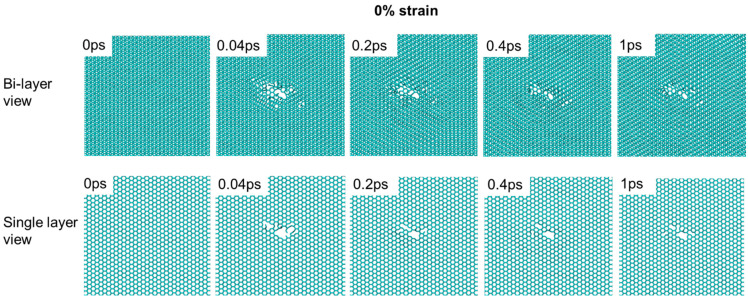
Damage cascade evolution in unstrained graphite, viewed as a bi-layer and as a single layer. The carbon knock-on energy is 3.7 keV.

The strain effect is not limited to point defect creation. The structural evolution under stress is also very different. Figure 7 shows the damage cascade evolution for a 10% strained sample, viewed either as a bi-layer or a single layer. The bi-layer view exaggerates the graphene cleavage around the damage cascade core. The cleavage leads to graphene plane gliding and different graphene packing. As shown by the bi-layer view, at 1 ps, regions around the cascade core have A-A packing while the other regions still exhibit A-B packing. The graphene plane develops cracking and local cleavage at the pore edge. In the single-layer view, a large cavity forms, with the edge decorated with a short chain-like one-dimensional segment. There is no noticeable defect recombination at the cavity. The cavity size increases with time. In comparison, Figure 8 shows both the bi-layer view and single-layer view of 0% strained graphite. In comparison to 10% strain, the cavity size is smaller, and there is no graphene gliding observed. The single-layer view shows that the cavity reduces its size from 0.04 ps to 0.2 ps. Overall, the 0% strain sample shows a certain capability of interstitial-vacancy recombination. The defect repair becomes much more difficult under 10% strain.

Using MD simulations to study the effect of stress on displacement and damage creation has certain advantages over first-principles calculations. First-principles calculations, in general, are more computationally costly. For atom-displacing process simulations, density functional theory (DFT) calculations and molecular dynamics simulations can be combined as DFT-based MD simulations. No such modeling results have been reported for graphite, but it is worth further study. When applying DFT calculations, long-range weak Van der Waals force needs to be appropriately included since Van der Waals force treatment has been an issue for DFT calculations.

## 5. Proton Irradiation and Raman Characterization

**Figure 9:**
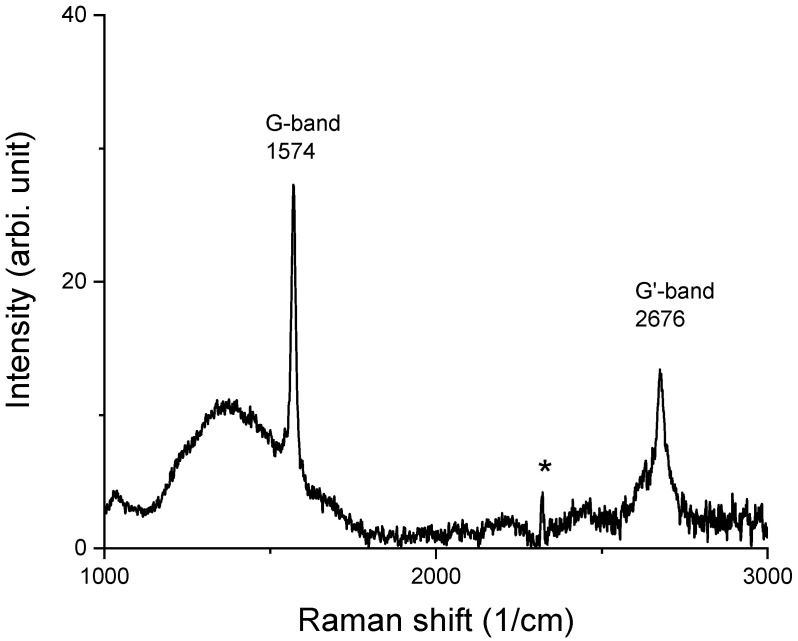
Raman spectrum from unirradiated HOPG.

The experimental studies included the steps of folding HOPG, irradiation on the folded edge, and Raman characterization. Figure 9 shows one typical Raman spectrum collected from unirradiated HOPG. The spectrum is featured with a G band at 1574 cm^−1^ and a G’ band at 2676 cm^−1^. The spectrum does not exhibit any sharp D band. Instead, there is a bump-like wide band centered around 1370 cm^−1^. Such a diffusive band is most likely caused by the existence of local amorphous zones from small cracks. As is shown, the D band from the irradiation-induced defects is distinctive. The peak labeled with “*” was previously reported to be due to N_2_ gas in the air [11]. The G mode is a doubly degenerate phonon mode at the BZ center and is characteristic of sp2 carbon networks. The G’ mode is also called the 2D or D* mode [11]. The mode is symmetry-allowed and appears in disorder-free crystalline graphite. The G’ mode is sensitive to the stacking order of the graphene along the c axis of graphite. Both the D and G’ bands originate from a double resonance Raman process. However, different from the D mode, the G’ mode contains no information on the crystalline sizes and defective levels on graphene planes. Therefore, the G’ mode was not analyzed in the present study.

**Figure 10:**
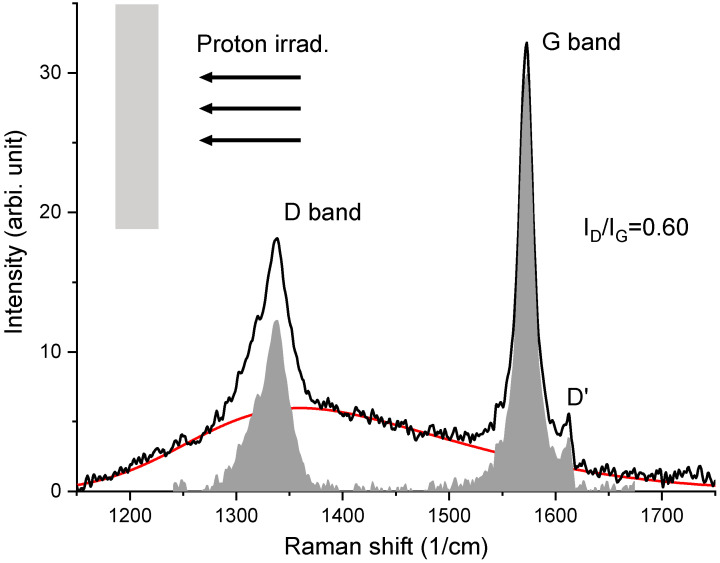
Raman spectrum from irradiated HOPG that was pasted on a flat surface (stress-free). The red line refers to the background used to extract D and G band intensities. The gray box represents the graphite substrate under irradiation. The arrows refer to the proton ion beam.

Figure 10 shows the Raman spectrum collected from an irradiated HOPG (stress-free). The HOPG was pasted on a flat surface. The side facing the ion beam was characterized by Raman. This sample refers to the condition without stress introduced. The G band appears at 1570 cm^−1^. A small D’ band appears at 1612 cm^−1^. The most significant change caused by the irradiation is the appearance of a sharp D mode at 1335 cm^−1^. The D mode is defect-activated and related to disorders such as kinks, vacancies, and impurities [8,9,10,11]. The irradiation-induced D band is very different from bump-like signals centered around the same region. Therefore, the Raman spectra are fitted using a skew-normal distribution function to separate the bump-like background from the spectra. The red line in Figure 10 is the best fit using

$$f\left(x\right)=cexp\left(-\frac{{\left(x-x_{c}\right)}^{2}}{\sigma^{2}}\right)\left[1+\mathrm{erf}\left(\frac{\alpha \left(x-x_{c}\right)}{\beta }\right)\right].$$

The gray-colored spikes are the *D* and *G* bands after subtracting the red-colored background. Both bands are integrated. The ratios of the integrated areas covered by each band are used to represent the ratios of the band intensities. We obtained $I_{D}/I_{G}=0.6$.

**Figure 11:**
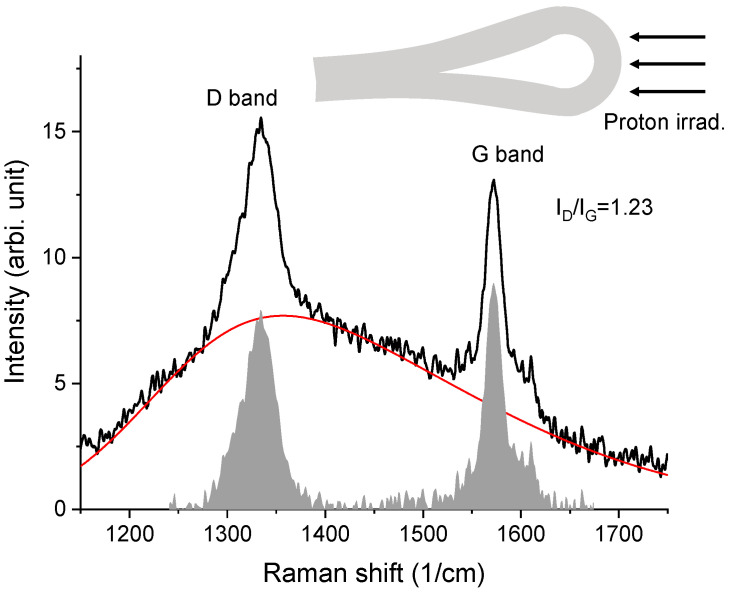
Raman spectrum from irradiated HOPG that was folded (experiencing tensile stress during the irradiation). The red line is the background used to extract the D and G band intensities. The schematics (gray) on the top represent the folded HOPG under ion irradiation.

For comparison, Figure 11 plots the Raman spectrum of the HOPG, which was folded during the irradiation. In order to remove the stress effect on the Raman characterization, the irradiated folded HOPG was flattened prior to the Raman characterization. The Raman spectrum was collected from the previously folded region. As shown in Figure 11, the spectral height at the D band becomes higher than that of the G band. After subtraction by the red-colored background line, the intensities of the D band and G band were calculated through areal integration. The $I_{D}/I_{G}$ ratio was measured to be 1.23, which is about a factor of two higher than the ratio in Figure 10.

**Figure 12:**
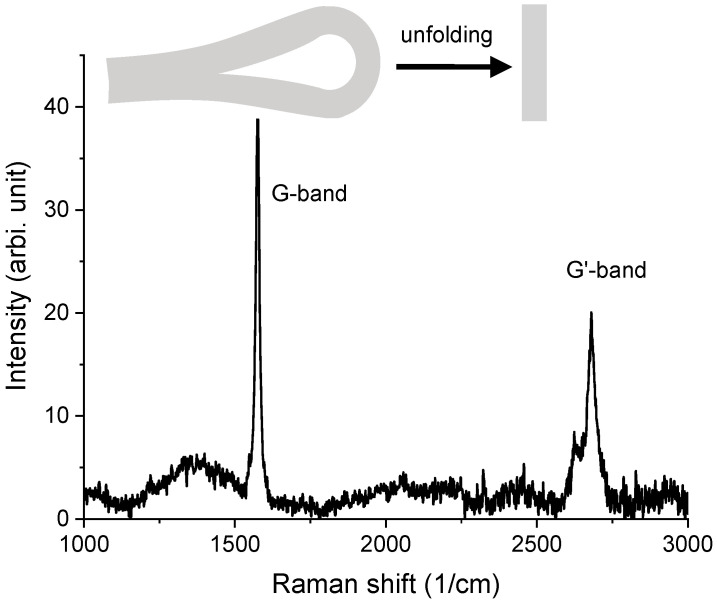
Raman spectrum from HOPG that was folded and then unfolded. No irradiation was performed.

The data in Figure 12 were collected from the HOPG that was folded and then unfolded. No irradiation was performed. The spectrum shows no D band ($I_{D}/I_{G}$ = 0). Compared to Figure 9 (virgin HOPG), Figure 12 shows that the Raman response of the folded → unfolded sample is similar to the virgin sample. In other words, there were no D band-related defects introduced by folding. This further suggests that the D band appearance in Figure 10 and Figure 11 was not caused by folding. Hence, $I_{D}/I_{G}$ is a valid indicator of radiation-introduced damage.

## 6. Discussions

The dramatically different $I_{D}/I_{G}$ ratios between the unstressed and stressed HOPG provide the evidence that tensile stress promotes defect creation in irradiation. Using the D/G ratio to quantitively determine the defect population is valid only when the defect density is not high enough to destroy six-fold rings. Based on the competing effect of the intensity enhancement by damage and intensity reduction by reduced six-fold ring numbers, the normalized $I_{D}/I_{G}$ ratio can be approximated by [8] the following equation:

(1) $$I_{D/G}^{*}=\left[\left(c_{1}\times dpa\right)+1\right]\times exp\left(-c_{2}\times dpa\right)$$

where “*” means the intensity is normalized by the unirradiated sample, which contains intrinsic defects. The factor $c_{1}$ is the constant describing the proportionality of intensity to the damage level. The exponential term $exp\left(-c_{2}\times dpa\right)$ describes the reduction of the six-fold ring density. The *D/G* trend changes as a function of damage levels were previously used to mark the onset of amorphization.

Using the SRIM code [31] and ${\bar{E}}_{d}\;$= 35 eV, as determined from the present study using the hybrid potential, the average damage level of the plateau damage zone of the proton-irradiated graphite (from the surface to about 37 microns) was 1.6 × 10^−3^ dpa. Note that the projected range of 2 MeV proton irradiation is 41 microns in graphite. The peak dpa was 3.8 × 10^−2^ but the damage peak region was too narrow in comparison with the plateau damage zone. Judging by the damage level of the plateau region (1.6 × 10^−3^ dpa), the proton damage level did not reach the critical level for amorphization. Hence, the enhancement in the D/G ratio reflects the enhancement in damage introduction.

In the modified K–P model [31], the number of Frenkel pairs is given by

(2) $$N_{d}=\frac{\kappa \left(E-\hat{Q}\right)}{2E_{d}}$$

where E is the total energy loss and $\hat{Q}$ is the energy loss due to electron excitation. κ is the displacement efficiency. The κ value is a constant independent of energy except for energy close to $2E_{d}$. It is approximately 0.8.

The present study shows that $\Delta E_{d}/\Delta s\%$ is a constant. In addition, the modeling shows that the changes are small. The linear approximation of Equation (2) around the zero strain point gives

(3) $$N_{d}\left(s\%\right)=\frac{\kappa \left(E-\hat{Q}\right)}{2E_{d}{}^{2}}\left(E_{d}+c\times s\%\right)$$

where s% is the tensile strain, $E_{d}$ is the threshold displacement energy at zero strain, and c is the slope of the $E_{d}$ vs. strain curve, which is 0.37 eV per 1% strain from the hybrid potential.

The present study shows that strain reduces the ${\bar{E}}_{d}$. The threshold displacement energy is determined by the energy difference between the lattice point and the saddle point in atom displacing. The changes in the bond length and inter-bond angles can both change energies. The distortion of the bond length and bond angles from the unstressed equilibrium configuration increases the energy of the carbon atom at the lattice side, leading to a reduced ${\bar{E}}_{d}$. This, however, is not the only mechanism in the damage enhancement. For the highest strain (10%), defect recombination is much less efficient than in other conditions. For other strains, the surviving defect number at 1 ps is proportional to the damage peak. However, 10% strain does not follow the same proportionality. Using 0% strain as the normalization factor, the peak damages are 1.17, 1.22, 1.35, and 1.41 for 2%, 5%, 8%, and 10% strain, respectively (as shown in Figure 6). The normalized surviving defect numbers are 1.16, 1.22, 1.43, and 1.77, respectively (also shown in Figure 6). The damage peak for 10% strain is enhanced by 41%. However, the final surviving defect number is enhanced by 77%. Such a large difference shows the difficulty in interstitial-vacancy recombination under the maximum strain.

## 7. Conclusions

Through a combined modeling and experimental study, we show that tensile strain reduces ${\bar{E}}_{d}$ and increases displacement numbers. The enhancement is linearly proportional to strain. At the highest strain, graphene gliding and local cleavage occur around damage cascades. The interstitial-vacancy recombination is less efficient under strain, leading to a second mechanism in defect enhancement. Experimentally, HOPG was folded to create tensile strain, and the folded region was irradiated by 2 MeV protons. Raman characterization showed the enhanced D/G ratio for the folded and irradiated region in comparison with the flat and irradiated region. A formula for the displacement estimation in strained HOPG is proposed based on the linear approximation. The strain effect needs to be appropriately considered in graphite applications.

## Data Availability

The data presented in this study are available upon request from the corresponding author.
